# Conservative Management of Giant Pyogenic Granuloma Post Strabismus Surgery: A Case Report and Literature Review

**DOI:** 10.7759/cureus.41321

**Published:** 2023-07-03

**Authors:** Mohammad S Bin Dlaim, Ghadah A Alhussein, Raneem S Alqahtani, Leenah T Almanea

**Affiliations:** 1 Pediatric Ophthalmology Division, King Abdullah bin Abdulaziz University Hospital, Riyadh, SAU; 2 College of Medicine, Princess Nourah bint Abdulrahman University, Riyadh, SAU

**Keywords:** conjunctival pyogenic granuloma, timolol, lobular hemangioma, capillary hemangioma, pyogenic granuloma

## Abstract

Ocular pyogenic granuloma is a benign vascular tumor that occurs primarily in children. Treatment options for pyogenic conjunctival granulomas include topical steroids, topical timolol, surgery, cryotherapy, and electrocautery. Patients with giant pyogenic granulomas are usually treated with surgical intervention. In this case, a 13-year-old Egyptian girl developed a giant pyogenic granuloma after strabismus surgery. Topical steroids showed a poor response and failed to demonstrate any improvement. While on timolol, the granuloma completely regressed, with no signs of recurrence. Despite the usual surgical approach to the treatment of purulent giant granulomas, we believe that topical timolol can be the preferred option as a noninvasive alternative therapy since it is considered safe when compared to the potential risks of topical steroid therapy or surgical exposure.

## Introduction

Ocular pyogenic granuloma (PG), also known as lobular capillary hemangioma, is a benign vascular tumor that occurs mainly in younger people and children [1]. There are two different terms to define the same lesion: 'pyogenic granuloma' is a misnomer because it is not associated with pus or granuloma formation but denotes a reactive condition, whereas 'lobular capillary hemangioma' is suggestive of a tumor [2].

These lesions often develop on cutaneous or mucosal surfaces [1]. The most commonly affected areas are the head, jaw, face, lips, palate, auricle, trunk, limbs, etc. [3]. It usually occurs as a result of inflammation associated with eye surgery for strabismus, trauma, or chalazion [1]. The exact etiology of ocular PG is unknown. Several factors have been pointed out as possible contributors to pathogenesis, such as trauma, hormonal effects, angiogenesis growth factor production, drugs, and viruses [4].

They are diagnosed in the clinical setting and confirmed histologically. They usually bleed or cause irritation or discomfort, prompting patients to seek treatment [5]. Treatment options for giant conjunctival PG include topical steroids, surgery, cryotherapy, and electrocautery [5,6]. Recent reports have shown that ocular and cutaneous PG can be resolved with the use of a selective β-blocker, topical timolol [5,6].

We report a case of giant conjunctival PG after strabismus surgery that completely regressed after topical treatment with timolol 0.5%. No adverse effects were reported.

This article was previously presented as a meeting abstract at the 7th Health Professions Conference on December 25, 2022.

## Case presentation

A 13-year-old Egyptian girl, medically free with the exception of strabismus, underwent uneventful strabismus surgery with bilateral medial rectus recession using polyglactin 910 (Vicryl) for partial accommodative esotropia.

Two weeks after surgery, the patient presented with mild redness at the surgical sites in both eyes. No history of trauma, itching, discharge, or rubbing was documented.

**Figure 1:**
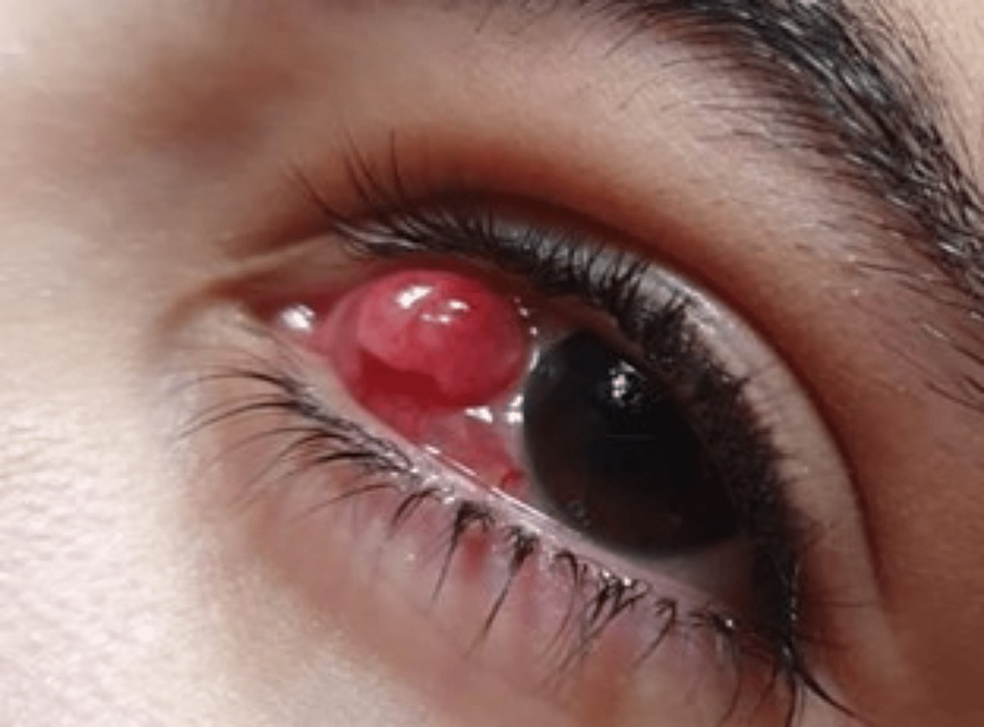
A 5*8*5 mm nasal conjunctival granuloma one month post surgery

One month after surgery, the patient presented with a giant conjunctival granuloma on the left eye. It was a red, congested, bloody, mushroom-like protrusion with an estimated base of 8 mm, height of 5 mm, and width of 5 mm, located in the nasal area of the left bulbar conjunctiva, and exhibiting a progressive pattern (Figure 1).

Eye examination revealed orthotropia with the prescribed glasses. Slit-lamp examination of both eyes showed a clear cornea with a deep anterior chamber.

**Figure 2:**
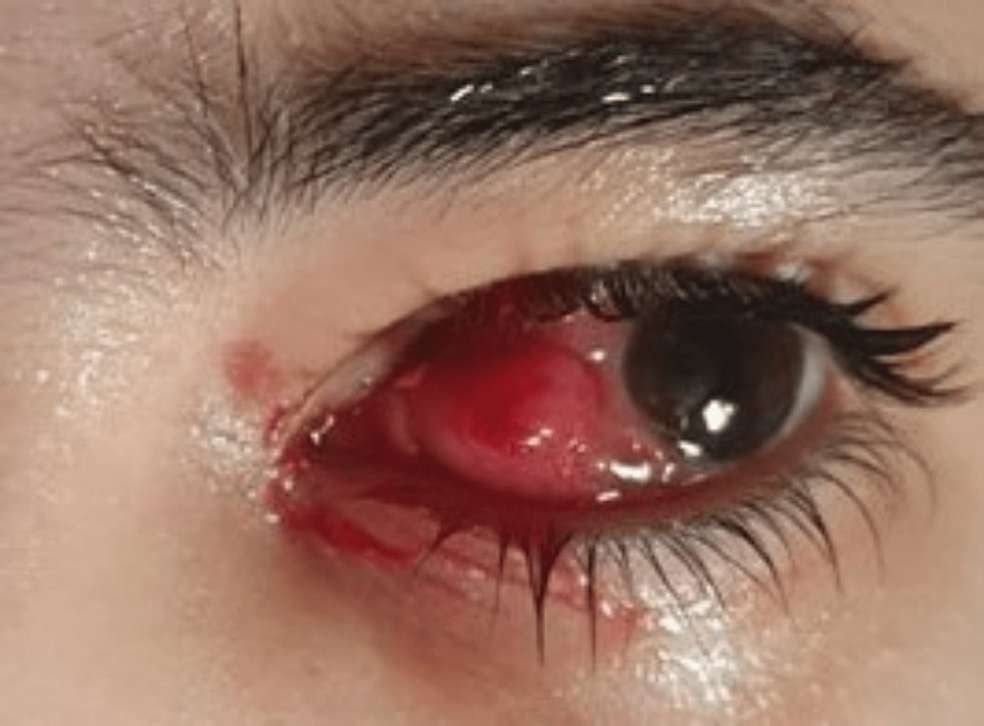
Poor response to extensive topical steroid therapy after four weeks

Post-strabismus surgery, the patient received 1% prednisolone acetate drops for the left eye every eight hours for two weeks, then the dosage was increased to two drops per hour, and then, she was switched to loteprednol etabonate 0.5% for two weeks once daily. No improvement was noted (Figure 2).

**Figure 3:**
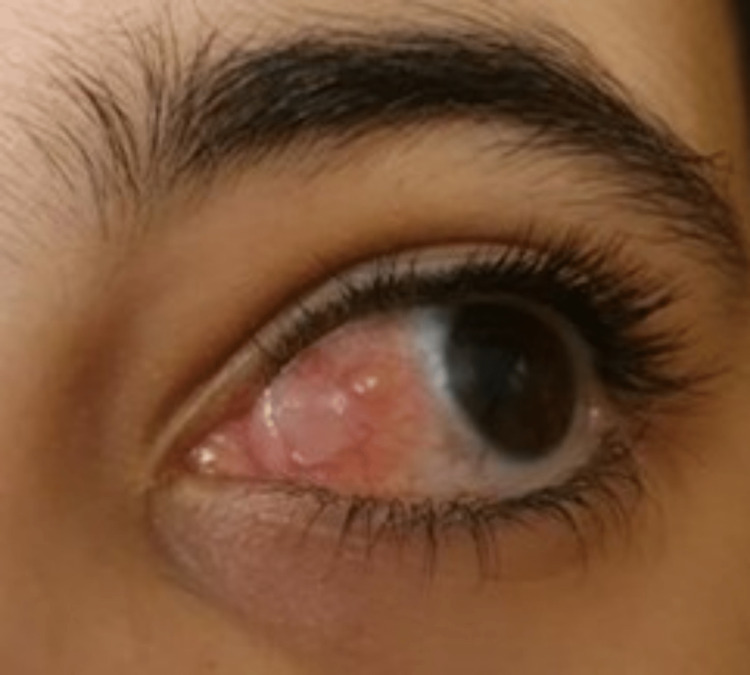
Remarkable regression of the granuloma after initiation of topical timolol at six weeks post surgery

Because of the constant progression of the granuloma, timolol maleate 0.5% was introduced to the left eye every 12 hours. After the introduction of timolol, the patient showed remarkable improvement, and the topical steroid was slowly discontinued (Figure 3).

**Figure 4:**
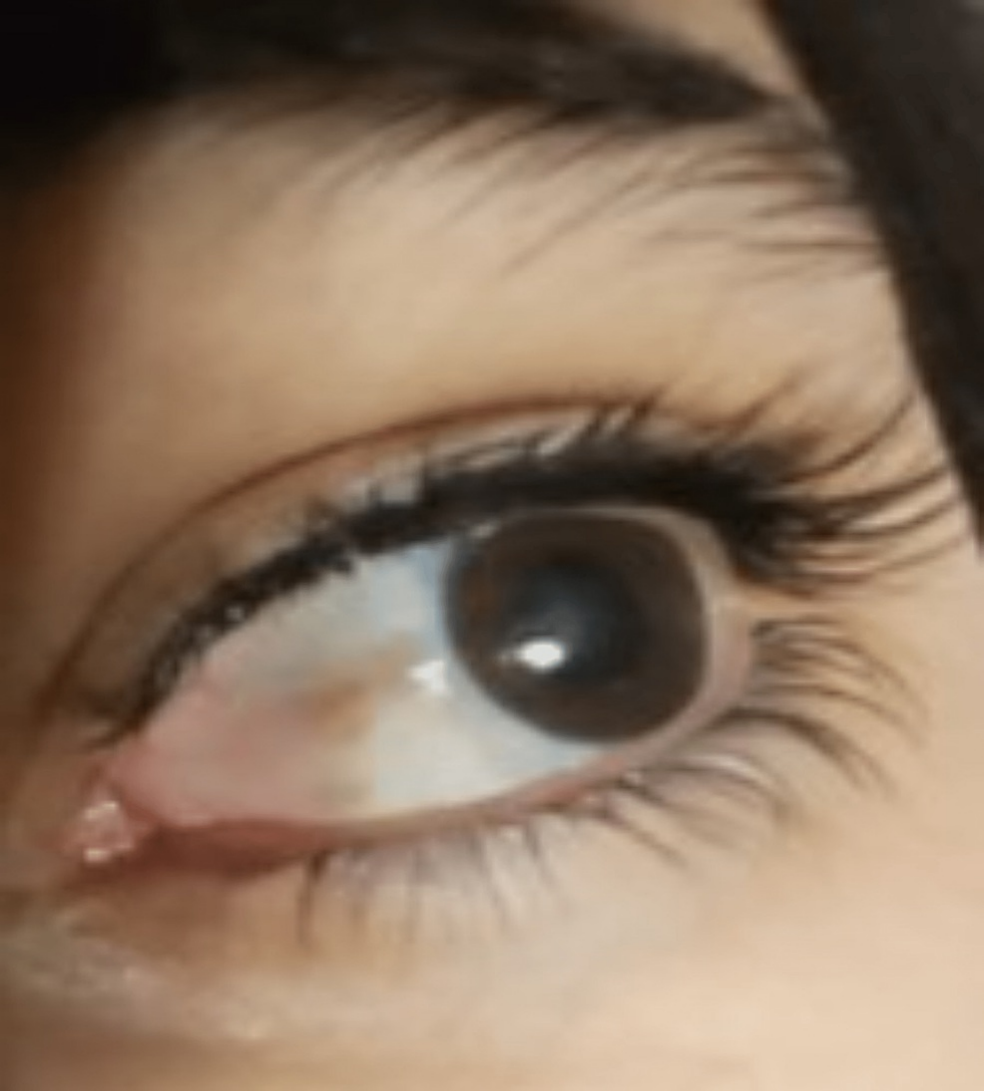
Lesion almost completely disappeared after 12 weeks

Complete regression was observed after three months following the surgery (Figure 4).

**Figure 5:**
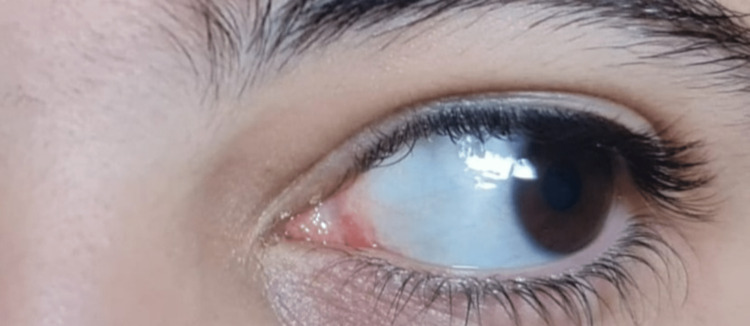
Complete resolution and no evidence of recurrence after 18 months post-surgery

Follow-up examinations were performed regularly at three months, six months, and 18 months; there was no evidence of recurrence (Figure 5).

## Discussion

The exact mechanism that causes pyogenic granulomas (lobular capillary hemangiomas) to develop is unknown. An imbalance in angiogenesis regulation is proposed as a mechanism involved in the formation of pyogenic granulomas [7]. To a certain extent, the previous process is linked to the proliferation and migration of inflammatory cells, vascular endothelial cells, and fibroblasts under the influence of cytokines, particularly the fibroblast growth factor [2]. Moreover, it is associated with impaired wound healing and vascular growth guided by Fms-like tyrosine kinase 4 (FLT4) and the nitric oxide pathway [8].

Furthermore, many factors, such as trauma, chalazion, or after ocular surgery, are believed to have a causal association with pyogenic granuloma [9]. A study that reviewed 100 cases of a pyogenic granuloma that involved the eye or its adnexa revealed that 42% of the cases were due to chalazion, while ocular surgery and accidental trauma represent 40% and 5%, respectively [9]. Conjunctival pyogenic granulomas are frequently encountered after surgeries for pterygium, strabismus, lacrimal duct obstruction, and enucleation [9,10]. Ocular granuloma formation is commonly associated with post-operative ocular surgeries using non-absorbable sutures. However, it has been reported that conjunctival granulomas can be caused by absorbable sutures [11]. Pyogenic granulomas with chalazion as a predisposing factor are more likely to arise from the conjunctival surface than the cutaneous surface. In 42 cases of pyogenic granuloma associated with chalazion, only 10 cases were preceded by surgical treatment for chalazion [9].

A pyogenic granuloma begins as a small, reddish papule, which progresses over weeks to months in size but does not grow beyond 1 cm [3,12]. Most clinic visits start at the stage where the lesion has stabilized with no spontaneous regression. If left untreated, the lesion might become sessile or pedunculated and might bleed extensively after minor trauma to the eye. The bleeding is thought to be recurrent and difficult to control if present [13,14].

By this, the diagnosis of pyogenic granuloma is straightforward, depending on the clinical presentation. It commonly presents externally at the palpebral conjunctiva, along with a history of a dome-shaped papule or erythematous with or without bleeding over a couple of days or weeks [13-15].

Further histopathologic examination is essential to exclude conditions that mimic pyogenic granuloma. Using hematoxylin and eosin (H&E) stain on the obtained section shows the formation of lobulated capillaries with fibrous septation and non-granulomatous inflammatory cells surrounded by edema [9,15].

Even though some individuals may have spontaneous healing, pyogenic granulomas typically need treatment due to recurrent ulceration and bleeding. Treating pyogenic granulomas can be tricky, as patients respond differently to a variety of treatment modalities. The aim of the treatment is to prevent further growth, recurrence, and any potential bleeding that might occur [3]. Furthermore, it is noted as being desirable to remove the PG and send the obtained tissue for histopathologic analysis to rule out malignancies that have the ability to mimic PG [16].

There is no unanimity or agreement on the optimal management of pyogenic granuloma, but different approaches are practiced and used for its management. The selection of the treatment technique depends on different factors, including size, location, patient age, and risk of recurrence [3,12].

Treatment modalities have been prescribed in a retrospective study. A total of 19 different treatment regimens were used on 1162 cases of PGs. The most common method used is surgical excision, which consists of either shaved excision or curettage followed by laser therapy or full-thickness excision [3].

Complete excision is preferred in terms of preventing recurrence and obtaining a specimen for histopathological examination [17]. Surgical excision with thermal cauterization followed by corticosteroid drops was effective in 52 cases of conjunctival granuloma with no recurrence at six months of follow-up [18]. In addition, multiple case reports of conjunctival pyogenic granulomas were successfully treated with surgical resection [15,17,19]. However, preserving the integrity of the structure is a difficulty in any surgical intervention. Also, the need for general anesthesia in children makes it less preferable in certain situations. Accordingly, careful postoperative assessment is also essential [17,18]. The combination of cryotherapy and intraoperative mitomycin C may aid in preventing recurrence after surgical excision [20].

Sclerosing agents, such as polidocanol, are also being used as a modality for the treatment of pyogenic granulomas [21]. Non-surgical methods include cryotherapy with liquid nitrogen, intralesional agents, and topical agents such as topical Imiquimod or a non-selective beta-adrenergic antagonist that can be favorable. In some cases, using laser therapy with a pulsed dye laser (PDL) can also be beneficial [3,12,13]. Recently, subconjunctival bevacizumab has been shown to be safe and effective for the treatment of refractory and recurrent pyogenic granulomas [22,23].

The size of a granuloma can be efficiently curtailed with topical corticosteroid treatment. To anticipate the probable adverse effect, it is necessary to keep a careful eye on the intraocular pressure and taper the dose. If the lesion does not respond to corticosteroids, surgical excision and histological examination are suggested [6,24]. 

Topical timolol has been shown to be effective in the treatment of conjunctival pyogenic granuloma in a modest number of children and adults without major side effects [25]. There have been several hypothesized proposals for how beta-blockers work in pyogenic granulomas. Peripheral vasodilation, stimulated expression of vascular endothelial growth factor and basic fibroblast growth factor, and prevention of endothelial apoptosis in cells are all mediated by beta-adrenergic receptors found on vascular endothelial cells [4]. Beta-blockers act by causing vasoconstriction in the lesion, causing its size to decrease. In addition, the inhibitory effects on vascular growth factors and the encouragement of apoptosis aid in regression [4]. Limited research has been done on the use of beta-blockers other than timolol for ocular pyogenic granuloma. However, propranolol has been shown to reduce the size and symptoms of other vascular tumors, such as infantile hemangiomas, although its efficacy and safety in the ocular context have not been extensively studied [25,26].

Topical timolol has been used safely; however, a significant reversible reduction in intraocular pressure was reported using timolol, which resolved after the treatment course [25]. In addition, a case series of four children with ocular pyogenic granuloma responded to treatment with only topical timolol with no recurrence in a three-month period of follow-up [5]. It has a smaller risk of side effects than topical steroid treatments or other pharmacological or surgical treatments [5]. This may be the preferred treatment modality if verified in additional larger studies with extended follow-up and controls.

Table 1 provides a synopsis of the reviewed studies.

**Table 1:** Review of the available studies PG: pyogenic granuloma

Authors	Publication year	Study design	Population	Intervention	Outcome
Oke et al.	2017	Case series	Four children with ocular pyogenic granulomas	Topical timolol 0.5% twice daily for a period of at least 21 days	Complete resolution was achieved in every case during the course of treatment, without any recurrence for not less than three months.
Espinoza et al.	2005	Retrospective observational case series	17 cases of conjunctival pyogenic granuloma after strabismus surgery	Topical corticosteroids prednisolone acetate (1%), dexamethasone (0.1%) for 3-80 days. Surgical excision for those who failed medical treatment	Topical corticosteroids were effective in 90% of the patients, whereas the surgical excision was successful in those who were unresponsive to medical treatment. No available data in the follow-up period.
Tidake et al.	2021	Case report	A 10-year-old boy with lobulated conjunctival granuloma in the right eye.	Complete surgical excision	Successful excision of the lesion however follow-up results are not mentioned.
Kasturi et al.	2019	Case report	A 45-year-old male presented with a Recurrent Conjunctival Pyogenic Granuloma in the left eye for six months	Subconjunctival Bevacizumab (2.5mg/0.1ml) which was given twice with an interval period of two weeks	Follow-up after one year of the injection showed resolution, with a small pseudo-pterygium temporally.
Pham et al.	2019	Case report	Two eyes that were treated with pterygiectomies and amniotic membrane grafts developed pyogenic granulomas.	Injections of intralesional triamcinolone did not demonstrate any signs of resolution. Bevacizumab injections were used after the failure of triamcinolone.	Following intralesional injections of bevacizumab, the vascular lesions completely disappeared without experiencing any side effects after a month of its administration. No extensive follow-up data was mentioned.
Putri et al.	2021	Case report	A 69-year-old male had left eye conjunctival pyogenic granuloma associated with untreated eye stye	oral and topical corticosteroids with a tapering dose	significant improvements were observed as the lesion regressed in size after five weeks of treatment without recurrence.
Nair et al.	2020	Retrospective study	12 patients with conjunctival pyogenic granuloma. (Mean age of 31.1 years)	0.5% timolol maleate eye drops twice a day and followed up for eight weeks.	11 patients had complete resolution of pyogenic granulomas after a mean duration of treatment of around four weeks. One had persistent PG that was surgically removed after sub-optimal resolution at six weeks of treatment.
Jaiswal et al.	2020	Prospective study	The study included 40 patients with pyogenic granuloma, excluding those who had previously received treatment or had recurrent PG. The mean age was 23.5 ± 13.3 years	Topical timolol eye drops (0.5%) given twice daily for four to six weeks.	31 patients (77.5%) had an excellent response to treatment, and there were no reported cases of lesion recurrence.
Shi et al.	2021	Case report	An 11-year-old girl presented with a conjunctival mass.	The lesion was excised surgically. Histopathology showed a granulomatous capillary hemangioma.	Complete excision of the lesion with no recurrence after six months of follow-up.
Zhang et al.	2018	Retrospective study	52 eyes of 50 patients that developed conjunctival granuloma after pterygium surgery.	Surgical excision with corticosteroid drops for one week then tapered within two weeks.	Successful surgical excision of all lesions and no signs of recurrence after a follow-up of 6 months.
Ashok Kumar et al.	2020	Case report	A 23-year-old pregnant female (37 weeks of gestation) presented with a 5 mm mass in the palpebral conjunctiva with active bleeding.	Surgical excision for histopathology and cauterization of the base	Complete excision of the lesion with base cautery. However, there is no available data on follow-up.
Suman et al.	2020	Case report	A 31-year-old female with recurrent polypoid conjunctival granuloma.	The mass was removed by excision, and intraoperative application of mitomycin C 0.02% for 1 minute and cryotherapy using a double thaw technique of the peripheral conjunctival margin were performed.	One year after treatment, there were no signs of recurrence.

## Conclusions

In the present case, despite the usual surgical approach to giant pyogenic granuloma management, we believe that topical timolol can be the preferred option as a non-invasive alternative therapy for pyogenic granuloma with consistent follow-up and a good assessment of the case.

This approach is very safe in comparison to the possible risks of topical steroid therapy or surgical exposure, especially with general anesthesia.

## References

[1] DeMaria LN, Silverman NK, Shinder R (2018). Ophthalmic pyogenic granulomas treated with topical timolol—clinical features of 17 cases. Ophthalmic Plast Reconstr Surg.

[2] Kamal R, Dahiya P, Puri A (2012). Oral pyogenic granuloma: various concepts of etiopathogenesis. J Oral Maxillofac Pathol.

[3] Lee J, Sinno H, Tahiri Y, Gilardino MS (2011). Treatment options for cutaneous pyogenic granulomas: a review. J Plast Reconstr Aesthet Surg.

[4] Patra AC, Sil A, Ahmed SS (2022). Effectiveness and safety of 0.5% timolol solution in the treatment of pyogenic granuloma: a randomized, double-blind and placebo-controlled study. Indian J Dermatol Venereol Leprol.

[5] Oke I, Alkharashi M, Petersen RA, Ashenberg A, Shah AS (2017). Treatment of ocular pyogenic granuloma with topical timolol. JAMA Ophthalmol.

[6] Espinoza GM, Lueder GT (2005). Conjunctival pyogenic granulomas after strabismus surgery. Ophthalmology.

[7] Jordan DR, Brownstein S, Lee-Wing M, Ashenhurst M (2001). Pyogenic granuloma following oculoplastic procedures: an imbalance in angiogenesis regulation?. Can J Ophthalmol.

[8] Godfraind C, Calicchio ML, Kozakewich H (2013). Pyogenic granuloma, an impaired wound healing process, linked to vascular growth driven by FLT4 and the nitric oxide pathway. Mod Pathol.

[9] Ferry AP (1989). Pyogenic granulomas of the eye and ocular adnexa: a study of 100 cases. Trans Am Ophthalmol Soc.

[10] Wu D, Qian T, Nakao T (2017). Medically uncontrolled conjunctival pyogenic granulomas: correlation between clinical characteristics and histological findings. Oncotarget.

[11] Demir S, Ortak H, Alim S (2014). Severe conjunctival foreign body reaction caused by polyglactin 910 (Vicryl) suture material following strabismus surgery. Turk Oftalmoloiji Derg.

[12] Mills SE, Cooper PH, Fechner RE (1980). Lobular capillary hemangioma: the underlying lesion of pyogenic granuloma. A study of 73 cases from the oral and nasal mucous membranes. Am J Surg Pathol.

[13] Pagliai KA, Cohen BA (2004). Pyogenic granuloma in children. Pediatr Dermatol.

[14] Dillman AM, Miller RC, Hansen RC (1991). Multiple pyogenic granulomata in childhood. Pediatr Dermatol.

[15] Tidake PK, Jaiswal S, Wagh V (2021). Conjunctival pyogenic granuloma - a case report. J Evol Med Dent Sci.

[16] Jaiswal H, Patidar N, Shah C, Singh R, Jain E, Piyush P (2021). Topical timolol 0.5% as the primary treatment of ophthalmic pyogenic granuloma: a prospective, single-arm study. Indian J Ophthalmol.

[17] Shi C, Ren Y, Feng J, Guo W, Zheng X (2021). Conjunctival granulomatous capillary haemangioma in children: case report and review of the literature. BMC Pediatr.

[18] Zhang Z, Yang Z, Pan Q, Chen P, Guo L (2018). Clinicopathologic characteristics and the surgical outcome of conjunctival granulomas after pterygium surgery. Cornea.

[19] Ashok Kumar D, Thajudeen H, Agarwal A (2020). A rare case of symptomatic ocular pyogenic granuloma in pregnancy. Indian J Ophthalmol.

[20] Suman S, Kumar A (2020). Intraoperative mitomycin C and cryotherapy as adjunct therapy for recurrent lobular capillary haemangioma of conjunctiva. BMJ Case Rep.

[21] Li J, Wu C, Song D, Wang L, Guo L (2021). Polidocanol sclerotherapy for the treatment of pyogenic granuloma in children. Dermatol Surg.

[22] Kasturi N, Senthamizh T, Bheemanathi H, Babu KR (2019). Subconjunctival bevacizumab for recurrent conjunctival pyogenic granuloma. J Pharmacol Pharmacother.

[23] Pham R, Phu N, Pham A, Chow C (2019). Treatment of pyogenic granulomas with intralesional injections of bevacizumab. Invest Ophthalmol Vis Sci.

[24] Putri Satwika AA, Made Juliari GA (2021). Conjunctival pyogenic granuloma associated with untreated eye stye: a case report. Asian Journal of Research and Reports in Ophthalmology.

[25] Nair AG, George RJ, Natarajan S, Jain V (2020). Topical timolol for the treatment of conjunctival pyogenic granulomas. Outcomes and effect on intraocular pressure. Indian J Ophthalmol.

[26] Berk DR, Culican SM, Bayliss SJ (2013). Scleral hemangioma: case report and response to propranolol. Pediatr Dermatol.

